# Correction: Visible-light-mediated Minisci C–H alkylation of heteroarenes with unactivated alkyl halides using O_2_ as an oxidant

**DOI:** 10.1039/d2sc90163c

**Published:** 2022-08-22

**Authors:** Jianyang Dong, Xueli Lyu, Zhen Wang, Xiaochen Wang, Hongjian Song, Yuxiu Liu, Qingmin Wang

**Affiliations:** State Key Laboratory of Elemento-Organic Chemistry, Research Institute of Elemento-Organic Chemistry, College of Chemistry, Nankai University Tianjin 300071 People’s Republic of China wangqm@nankai.edu.cn; Collaborative Innovation Center of Chemical Science and Engineering (Tianjin) Tianjin 300071 People’s Republic of China

## Abstract

Correction for ‘Visible-light-mediated Minisci C–H alkylation of heteroarenes with unactivated alkyl halides using O_2_ as an oxidant’ by Jianyang Dong *et al.*, *Chem. Sci.*, 2019, **10**, 976–982, https://doi.org/10.1039/C8SC04892D.

The authors regret that the regioselectivity of product 36 in Table 3 was incorrect. The correct structure, a regioisomer of the originally proposed structure, is shown below.
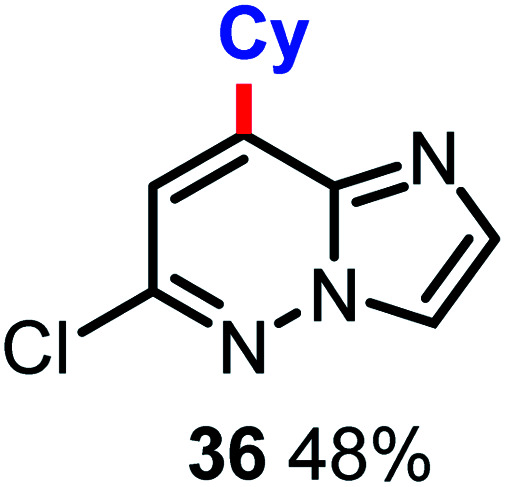


Table 3 within the original manuscript should therefore be as follows:

**Table:** Scope of the reaction with respect to the N-heteroarene^a^

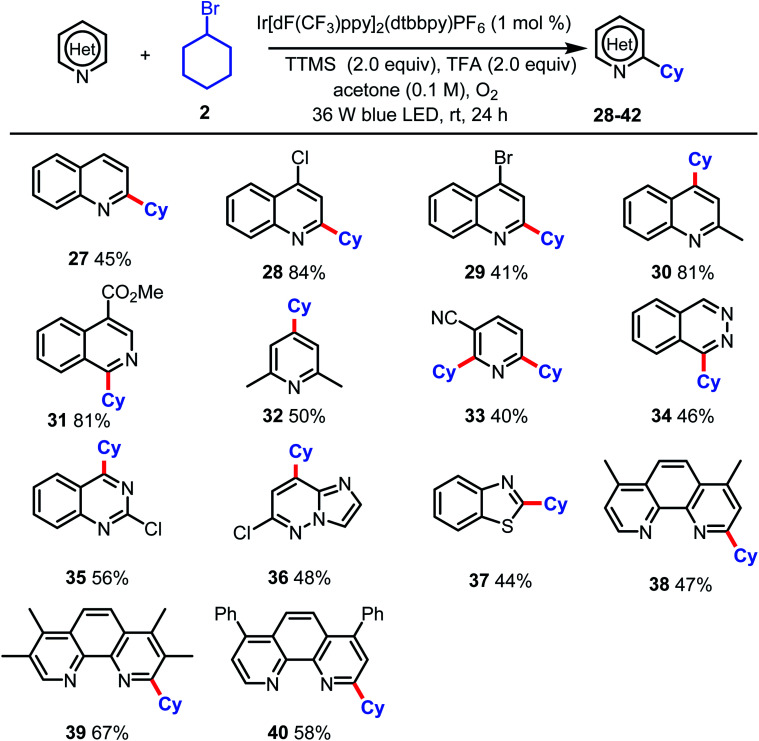

a Reactions were performed on a 0.3 mmol scale. Isolated yields are given.

The Royal Society of Chemistry apologises for these errors and any consequent inconvenience to authors and readers.

## Supplementary Material

